# Suggestions in Dental Records, Book-Keeping and Nominclature

**Published:** 1904-08-15

**Authors:** H. T. Smith

**Affiliations:** Cincinnati, Ohio


### SUGGESTIONS IN DENTAL RECORDS, BOOK-KEEPING AND NOMENCLATURE.

BY H. T. SMITH, D.D.S., CINCINNATI, OHIO.

Read before the Ohio State Dental Society, December, 1903, and printed from advance proofs by courtesy of "Dental Summary."

The season of this meeting occurring as it does in December, and just previous to the time when the dentist will give some attention to his dental records and account books, seems propitious, and is my excuse for offering some suggestions upon the subjects of dental records, nomenclature and book-keeping.

In regard to nomenclature: I have, for some time, thought that our words for expressing and for recording the degree of penetration of caries in tooth substance have been somewhat deficient. In attempting to accurately record these stages of dental caries I find that we have practically only two terms, the words "superficial" and "deep seated." To indicate the other stages with more or less involvement of the pulp, the use of entire phrases and sentences are necessary, as "pulp nearly exposed," "pulp quite exposed," etc. The profession need not be so impoverished of ideas, and especially in regard to dental caries, the treatment of which claims the major portion of the dentist's time. The disease certainly deserves the most complete and definite nomenclature possible.

As a precedent, what have we for instance in general therapeutics? There we find a certain prescription for the burns of the first degree, and a different prescription for the burns of the third degree, indicating that there is a definite nomenclature for burns; and yet the demarkation between those degrees is not *more* definite than between the different stages of dental caries that we might formulate.

A definite dental nomenclature seems to date back to the time of the World's Columbian Dental Congress in 1893, when Doctor Black presented his essay upon that subject, and he calls attention to the fact that there was at that time in use among the French dentists a nomenclature of dental caries. A modification of that scheme, according to Dr. Julio Endelman, is the one at present in use in France and elsewhere on the continent, and it is the use of this nomenclature that I would suggest for adoption.

The scheme is briefly the classification of the penetration of dental caries in tooth substance into four principal degrees, as follows:

Caries of the *first degree* is that in which the enamel has become decalcified, the disturbance being limited to the enamel tissue. Indicated caries 1°.

Caries of the *second degree* is that which affects the enamel and dentin, the pulp being as yet protected by a sound layer of dentin. Indicated—Caries 2°.

Caries of the *third degree* is that in which the carious process has penetrated to the extent of nearly exposing the pulp, this being coverned by a softened and infected layer of dentin. Indicated—Caries 3°.

Caries of the *fourth degree* is that in which there is an actual exposure of the pulp of a greater or less extent, including all the stages of inflammation and putrifaction of that organ. Indicated—Caries 4°.

Paul Dubois has further subdivided the fourth degree of dental caries into five different stages, designated as $4^1$, $4^2$, $4^3$, $4^4$, $4^5$ Thus:

$4^1$. Pulp disturbances of a slight degree, causing pulpitis.

$4^2$. Infection of the canals, without affecting the pericementum.

$4^3$. Infection of the dentinal canals and apical space, causing slight pericementitis, subacute or chronic.

$4^4$. Infection producing slight alveolar disorders, gingival fistula, or alveolar abscess of moderate intensity.

$4^5$. Infection producing serious disorders, acute or chronic pericementitis, with suppuration, pericemental cysts, and reflex disorders.

The advantages of such a system would be several. In our dental schools it would facilitate descriptions and enable the student to associate recognized forms of treatment for different stages in the progress of dental caries. In the papers and discussions of our dental societies the same advantages would obtain, increasing the facility with which they could be understood by ourselves and by foreign dentists; and apropos of the subject in hand, the increased facility with which we could record operations and transscribe history of cases, would be quite worth while.

The system of nomenclature just presented might be changed in some of its details, and modifications would perhaps suggest themselves to us all, but with a little study it will be found, that, on the four principal degrees, the stages of penetration in the progress of dental caries are concisely covered.

The further division of the fourth degree is somewhat arbitrary, but it seems to cover the various stages of pulp infection and sequelæ.

If we aim at universality in dental nomenclature, and that should be our object, the scheme could be used as it is with advantage.

There is one other suggestion in nomenclature which might be made—that is, the adoption of the words "currette" and "curretting" into our dental vocabulary. If I remember rightly, Dr. M. L. Rhein mentioned the term a year or more ago. I would suggest that "curretting" be made to indicate that part of the surgical treatment of pyorrhea alveolaris which consists in the scraping of the roots of teeth. It might include the process or removal of calcareous deposits from any part of the teeth, but perhaps better, should be confined to the instrumentation of the roots. That would distinguish the ordinary removal of calculus with the scalers above the gingival lines in the process of cleaning teeth from the more thorough scraping or curretting of the roots in the treatment of pyorrhea. We would then have a distinct class of pyorrhea instruments known as dental currettes—the Allport Currettes, for instance. In our dental records, the term curretting would be useful as indicating a definite operation.

And finally, the book-keeping system which I have to present is briefly the adaptation of the loose-leaf ledger system, now so commonly used in mercantile houses, to the needs of the dentist.

The diagram of the teeth is one that I have revised, and to the records commonly kept I have added a space for "Saliva Test" and one for "Temperament" and a space for "History of the Case."

The page is nine and one-half by eleven and one-half inches in size and is ruled with a double column for accounts, giving about sixty lines.

The W. B. Carpenter Co., of Cincinnati, have made this copy for me and will make additional copies for any dentist who will send for them.

DISCUSSION.

Dr. S. D. Ruggles: The offering without copyright of a system for records by a busy man indicates a high regard for professional ethics, and I have nothing but commendation for Doctor Smith in this most neglected field.

Dental nomenclature has been perfected to a very high degree, especially through the efforts of Doctor Black, and by its help we can designate with great accuracy the angles, surfaces and lines of teeth and cavities; but how many could stand before this body and tell in one word the exact degree of penetration of caries in a given case conveying the same idea to every one present?

Doctor Smith's suggestions are similar to a system offered the profession some years ago by a French author, at the Columbian Congress at Chicago, and published in its proceedings. The latter, however, had too many subdivisions and offered additional opportunity for error.

I find it quite a burden at times to transfer records from my Allen blanks to my ledger, for the average assistant cannot be relied upon, and the laws of the State accept none but the original record.

The card system idea the Doctor has incorporated with his scheme is a great saving of time.

You will notice that no provision is made for the records of deciduous teeth, therefore, I would suggest the addition of a small diagram in a different colored ink.

Dr. J. R. Callahan: I have paid some attention to the card system and to this form of book-keeping, and I believe these are the best diagrams and past-account sheets I have ever seen. I think it is the best of its kind. I don't know, but I shall try it, although I am wedded to the card system for many reasons. The Doctor spoke of making a record of your cases on a slip, and handing that to your book-keeper, or lay it on the desk. That becomes your original account, and when you go to the court, and the court asks for the original account, you haven't got it, and your ledger cannot fill the bill. So it has been my practice to make my ledger account the original account, and I can swear to that in court. It seems to me that that is an important item.

DR. JONES: There are just two things I follow—that is a place allowed for registering the temperament, and the condition of the saliva. We all know that the decay is only a local evidence of the condition of the patient's health, and it is a nice thing to know what the saliva test would be at this year and what would be the experience a year later. Probably the conditions are altogether changed, and you would alter your treatment.

DR. W. H. TODD: I think a system of this kind is almost absolutely necessary in dental practice. I am wedded to the card system, and I want to speak of a little thing I have in my office. I found, when I went to Chicago last year, that the dentists there had a pad upon their table, and the young lady in the office would write upon that pad the name of every person who enters the office, whether it be a collector, or a visitor, or some one to see the young lady herself, or some one to see the dentist, and it is the nicest thing I ever had in my office. I found when I came home in one of our stores here a book that had every day divided off—every day in the year. The book is left on the desk, and the young lady opens it at the day of the month, and it has, of course, the year, because it is just a year book, and every person who enters my office she puts his name down, and not only that, but the time he came into the office, so that if there is a dispute about the time you have the record there. If there is a telephone message it goes down, and at the end of the day I can look over this, and if I have missed any charges, if I have missed any telephone messages, or missed somebody, I find it right there—there is a record of it, and I know of many times I have saved twice the price of the book in one week by finding out that there was a treatment I didn't charge for. I think it is one of the nicest things I ever had.

Dr. Wilson: This little book that Dr. Todd has presented may have another good feature about it. There has been quite a good deal of thieving around dental offices and if every person who comes in there is put down, there may be some chance of detecting or finding out where these thieves are.

Member: I am charmed with Dr. Jackson's method of recording. He uses this arrangement when a patient comes in: In his reception-room he has a desk at which he has a stenographer, and while he is talking about the case the stenographer takes down the conversation, at least the salient points of it, and it is a permanent record, and could be used if necessary. It seems to me there is a great deal of misunderstanding sometimes as to price, etc., and many other things we might think of that would be proper in a record.

Dr. Barnes: We all like the idea of keeping a record in such a way that it would be valuable to us in years hence. The only thing I would suggest in taking saliva tests is that the time of taking test be given, whether in the early morning, or noon, or whether after dinner, or just as the business of the day is closed, because there is a difference, and we should keep a record of this. I think that the dot system of general record is good, but whether it would make this more valuable than it is you can judge, but I think it would, because you would be able to place upon one line all the operations of a single side; whereas, if you had three or four different operations you would have three or four different lines covered. You could have places for gold—and it is well to put down the kind of operation you perform, because you want to know for all time to come how long certain fillings have lasted in certain mouths, and how often decay is recorded. If you have any space at the right of the card for remarks, you can use it. I have on my card which I have changed some from the dot system—I have D. C. F., which indicates the pulp was destroyed and the canal filled. I have A. B. C. F., which means abscess and canal filled. You can refer back to this part and you have no trouble. I have each operation as a particular schedule, and if patients ask me the number of operations, I have it there for the last ten or fifteen or twenty-five years. I have the original record on a pad attached to a book. My assistant takes that from the pad which is bound in stiff covers, and in this book is placed the time when that was commenced, and the time it closed, and she makes the record when the day's work is done, and when that book is full, those are filed away so that we have no trouble at getting at the original record, and there is no reason why it should not be adopted in the card system.

Dr. Smith: There is only a word to say in closing the discussion, and that is in regard to the system of nomenclature. I will say that it appeals most strongly to those men who are having daily duties in dental colleges. In their efforts to make definite statements to students, to have them follow definite lines of treatment, and for getting indications of disease, I think it would appeal very strongly. Personally, I shall make an effort to have this adopted as part of the Black system of nomenclature. There is no reason why the American dentist should not have a system.

In regard to original accounts, the statement was made that the transferred account was not valuable in the court. That is true. It is the original entry, so-called, which would be good evidence, and the idea I had was to make this the original entry and this would be valid before any court of law. This is nicely and well bound, and it could be filed away with proper dates, and there is no reason why we should not have the data that might be asked for in a court.

A dentist can probably, at the end of his day's work, remember just what he has done, and what particular cavities were filled, and the condition of the patient, and there is no reason why he could not make an original entry in the ledger that will become the best evidence in a court.

But it seems to me that to men who are working without assistants to make the records, this is a nice way of making records, including the charges for the individual operation. The diagram being very much like that in the book, gives an opportunity to transfer it exactly. My idea was that such a pad as that to make the charge on could be counted an original entry.

Any method which a dentist has already adopted for keeping accounts could be brought into use with this system.

